# Genome-wide association study identifies multiple susceptibility loci for craniofacial microsomia

**DOI:** 10.1038/ncomms10605

**Published:** 2016-02-08

**Authors:** Yong-Biao Zhang, Jintian Hu, Jiao Zhang, Xu Zhou, Xin Li, Chaohao Gu, Tun Liu, Yangchun Xie, Jiqiang Liu, Mingliang Gu, Panpan Wang, Tingting Wu, Jin Qian, Yue Wang, Xiaoqun Dong, Jun Yu, Qingguo Zhang

**Affiliations:** 1 Chinese Academy of Sciences and Key Laboratory of Genome Science and Information, Beijing Institute of Genomics, Chinese Academy of Sciences, Beijing 100101, China; 2 Department of Ear Reconstruction, Plastic Surgery Hospital, Chinese Academy of Medical Sciences, Beijing 100144, China; 3 Department of Anatomy and Cell Biology, Brody School of Medicine, East Carolina University, Greenville, North Carolina 27834, USA; 4 Department of Cardiology, Beijing Anzhen Hospital of the Capital University of Medical Sciences, Beijing 100029, China; 5 Beijing KPS biotechnology, Beijing 102206, China; 6 Department of Internal Medicine, College of Medicine, The University of Oklahoma Health Sciences Center, Oklahoma City, Oklahoma 73104, USA

## Abstract

Craniofacial microsomia (CFM) is a rare congenital anomaly that involves immature derivatives from the first and second pharyngeal arches. The genetic pathogenesis of CFM is still unclear. Here we interrogate 0.9 million genetic variants in 939 CFM cases and 2,012 controls from China. After genotyping of an additional 443 cases and 1,669 controls, we identify 8 significantly associated loci with the most significant SNP rs13089920 (logistic regression *P*=2.15 × 10^−120^) and 5 suggestive loci. The above 13 associated loci, harboured by candidates of *ROBO1*, *GATA3*, *GBX2*, *FGF3*, *NRP2*, *EDNRB*, *SHROOM3*, *SEMA7A*, *PLCD3*, *KLF12* and *EPAS1*, are found to be enriched for genes involved in neural crest cell (NCC) development and vasculogenesis. We then perform whole-genome sequencing on 21 samples from the case cohort, and identify several novel loss-of-function mutations within the associated loci. Our results provide new insights into genetic background of craniofacial microsomia.

## Abstract (web summary)

Craniofacial microsomia is a congenital anomaly that affects the development of the skull. Here, the authors perform a genome-wide association study on patients in China and identify particular loci that provide insights into genetic mechanisms.

## Graphical abstract

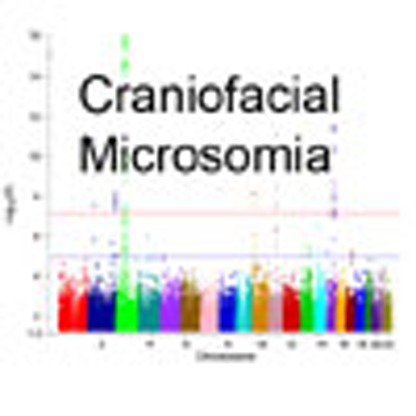

Craniofacial microsomia (CFM, MIM: 164210) encapsulates congenital anomalies of the external and middle ear, maxilla, mandible, facial and trigeminal nerves, and surrounding soft tissues on the affected side^1^. The occurrence of CFM is between 1 in 3,000 and 1 in 5,600 living births^2^. Popular assumptions for the pathogenesis of CFM include neural crest cell (NCC) disturbance and vascular disruption^3^. The NCCs originate from the neural ectoderm, migrate over long distances to participate in the formation of the first and second pharyngeal arches, and give raise to craniofacial structures^4^. Mouse models have indicated that dysfunctional genes involved in NCCs delamination, proliferation, migration or reciprocal interactions with other cell types in pharyngeal arches would cause impairments of the craniofacial development^5^. Through vascular disruption during the morphogenesis of the craniofacial vascular system^6^, localized ischaemia has been considered as another risk factor for CFM, although this notion is debatable^7^.

Many studies have revealed that CFM is caused by inherited and/or environmental factors^3^,^8^,^9^. Genetic variants are largely believed to contribute to this anomaly. Despite that various CFM candidate genes were proposed from mouse models or human syndromes with CFM^3^, to date, very few genetic variants have been identified and validated in human.

To fill in gaps in our knowledge about CFM and to decipher its genetic basis, we perform the first genome-wide association study (GWAS) along with whole-genome sequencing (WGS) in CFM patients from China. We find eight significant and five implicated loci associated with CFM. Functional analyses on these loci identify multiple CFM candidate genes involved in NCC development.

## Results

### Basic GWAS results

For discovery, we conducted a GWAS in 939 CFM cases and 2,012 healthy controls from China, by testing single-nucleotide polymorphisms (SNPs) that satisfied quality control (792,342), with or without stratifications on subgroups of gender and (left- versus right-) side-affected CFM. We then evaluated the significant SNPs with a *P* value <1 × 10^−5^ from the discovery stage in validation set of 443 cases and 1,669 controls from China.

**Figure 1:**
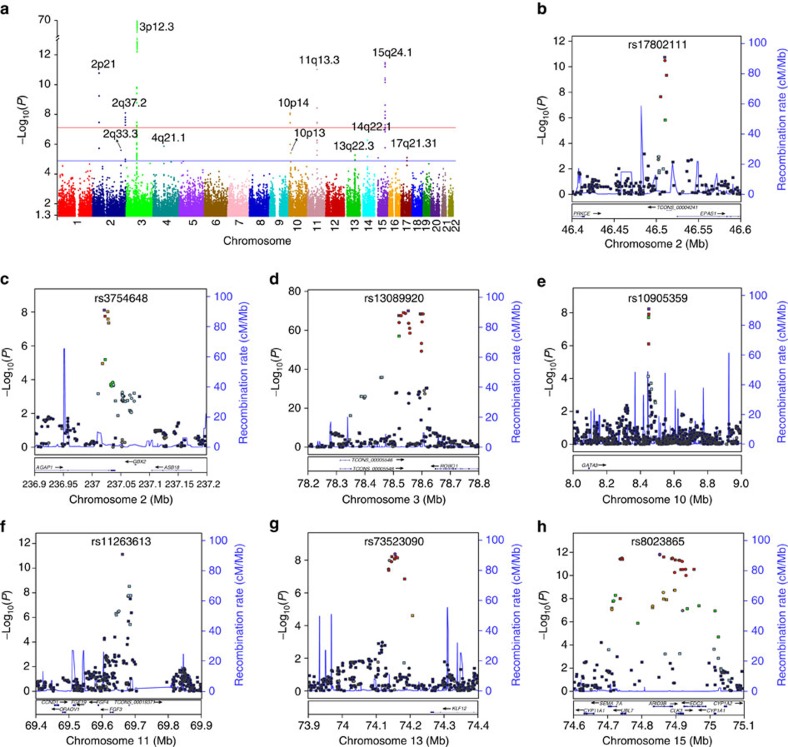
Manhattan plots of the *P* values calculated from the genome-wide association study at the discovery stage. (**a**) Data were collected from 939 cases with craniofacial microsomia and 2,012 controls on 792,342 SNPs that had passed the quality control. The −log_10_(logistic regression *P* value) of each SNP is shown as a function of genomic position on the autosomes (hg19). Genome-wide significance (solid red line; *P*≤6.3 × 10^−8^) and suggestive significance (solid blue line; *P*≤1 × 10^−5^) are denoted. (**b**–**h**) Regional plots shows the association of craniofacial microsomia risk with all significant loci, continuous genomic regions surrounding the lead SNPs, including 2p21 (**b**), 2q37.2 (**c**), 3p12.3 (**d**), 10p14 (**e**), 11q13.3 (**f**), 13q22.1 (**g**) and 15q24.1 (**h**). Each point represents a SNP plotted with its −log_10_*P* value as a function of genomic position (hg19). Imputation analysis is shown with circles and direct genotyping with squares. In each regional plot, the purple symbol denotes the lead SNP, showing its name on the top of each plot. The colour coding of the rest of the SNPs showed in LD with the lead SNP: red, *r*^2^≥0.8; gold, 0.6≤*r*^2^<0.8; green, 0.4≤*r*^2^<0.6; cyan, 0.2≤*r*^2^<0.4; blue, *r*^2^<0.2; grey, *r*^2^ unknown. Recombination rates were estimated from ASN population of 1KG project (Mar 2012). Gene annotations were taken from the UCSC genome browser.

Logistic regression (LR) analyses on the two combined sample sets identified seven genome-wide significantly (*P*<6.3 × 10^−8^, the Bonferroni-corrected significance threshold) associated loci with lead SNPs of rs13089920 (LR *P*=2.15 × 10^−120^, odds ratio (OR)=5.18), rs10459648 (LR *P*=2.86 × 10^−23^, OR=0.63), rs17802111 (LR *P*=9.57 × 10^−18^, OR=1.48), rs11263613 (LR *P*=7.91 × 10^−17^, OR=1.68), rs3754648 (LR *P*=6.33 × 10^−13^, OR=1.39), rs7420812 (LR *P*=6.74 × 10^−10^, OR=1.33), and rs10905359 (LR *P*=5.11 × 10^−9^, OR=0.76; Fig. 1, Table 1). In addition, five implicated loci with lead SNPs of rs3923380, rs754423, rs4750407, rs9574113 and rs7222240, reached a suggestive genome-wide significance level (LR *P*<1 × 10^−5^; Table 1, Supplementary Fig. 1). LR analyses on the subgroups of CFM on gender and affected-side identified a significant associated locus with a leading SNP of rs17090300 (LR *P*=1.04 × 10^−11^, OR=2.31) to left-side-affected CFM patients (*n*=481; Supplementary Fig. 2). The significant heterogeneity of association pattern (Cochran's *Q*-test *P*=2.09 × 10^−6^) was found between the left- and right-side-affected subgroups at rs17090300 (Supplementary Table 1). The phenotypic variance explained by the significantly associated and implicated lead SNPs were 6.92% and 1.96%, respectively, with a prevalence rate of 1.4 per 10,000 in China^10^. Furthermore, the joint effect of all the 792,342 genotyped SNPs could explain 28.4% of the variance observed in this study.

### Imputation followed by conditional and joint analyses

To identify additional associated variants, we imputed the untyped variants from genotyping data and the haplotype information provided by the 1000 Genomes Project (1KG). Among the imputed variants, we identified 68 additional SNPs (LR *P*<1 × 10^−5^) associated with CFM risk in the 13 associated loci (7 significant, 1 left-side specific, and 5 suggestive loci, Supplementary Data 1). To assess whether the 68 SNPs were independent from our initially identified leading SNPs, we performed conditional analyses on the genotyped and imputed variants. We did not find any other independently associated variants (Supplementary Fig. 3). To identify other new loci associated with CFM, we used multiple regression (by Wu *et al*.^11^) to test for the joint effect of the variants from a gene or haplotype block. We were able to replicate some of the identified loci, and did not find additional associated ones (Supplementary Table 2). Thus, no more new associated variants or loci were identified in conditional and joint analyses.

### Functional annotation and eQTL analysis

Functional non-coding variants within gene regulatory elements may potentially result in a disease phenotype through modulating gene expression level. To predict the effects of variants on gene expression, we submitted 291 SNPs (including 151 imputed SNPs) with a *P* value <1 × 10^−4^ to SeattleSeq Annotation 138 and HaploReg (v2) for analyses^12^. We found six SNPs located in known transcription factor-binding sites (TFBS), and three of them (GWAS *P*<6.3 × 10^−8^) located near or within *ROBO1* (rs147642420), *KLF12* (rs7986825) or *ARID3B* (rs7497036) (Supplementary Data 2). Among the HaploReg annotated variants, 187 SNPs were located in gene expression regulatory motifs, such as the enhancers, promoters, open chromatins and protein-binding sites (Supplementary Data 3). For enrichment analyses of cell type-specific enhancers and DNase hypersensitive sites, we conducted queries in HaploReg with the 291 SNPs and their linked SNPs (*r*^2^=1), based on the epigenomic data from ENCODE or Roadmap. As for ENCODE data, these SNPs were enriched in the enhancers or DNase hypersensitive sites of eight cell lines (Supplementary Data 3), noting that the fold change from observed to expected ‘strongest enhancer' was 15.6 (binomial test *P*<1 × 10^−6^) in H1 embryonic stem cells. As for Roadmap data, these SNPs were significantly (*χ*^2^-test *P*=1.3 × 10^−4^) enriched in the enhancers of stem cells and stem cell-derived cell lines (Supplementary Fig. 4).

To confirm the relations between CFM-associated SNPs and gene expression, we used Genevar to map the expression quantitative trait loci (eQTL) by correlating the SNPs with gene expression levels in lymphoblastoid from HapMap populations^13^. We found that several lead SNPs or their linked variants (*r*^2^>0.8, calculated from Asian populations of 1KG) had nominal associations (*P*<0.05) with the expression levels of the nearest genes (Supplementary Data 4), such as *ROBO1* (rs4401330, linear regression *P*=9.2 × 10^−3^, in CHB), *KLF12* (rs7986825, linear regression *P*=8.8 × 10^−3^, in GIH), *EDNRB* (rs5351, linear regression *P*=5.1 × 10^−3^, in YRI) and *SHROOM3* (rs4859453, linear regression *P*=2.0 × 10^−3^, in JPT). We then looked into the regulatory function of these nominal *cis*-eQTLs and found that 63% of them were located within the promoters, enhancers, DNase hypersensitive sites or TFBS (Supplementary Data 5). Enrichment analyses for these regulatory elements showed that embryonic cells, epithelial cells and carcinoma cells were significantly enriched for those ‘strongest enhancers' or DNase hypersensitivity sites.

### Pathway analyses

To identify the CFM candidate genes from the 13 associated loci and their potential connections, we used Gene Relationships Across Implicated Loci (GRAIL) methods^14^ to analyse the 46 genes within the 13 associated loci (Supplementary Data 6). Overall, 13 candidate genes were identified by GRAIL as follows: *ROBO1*, *GATA3*, *EPAS1*, *PARD3B*, *GBX2*, *SHROOM3*, *FRMD4A*, *FGF3*, *KLF12*, *EDNRB*, *NID2*, *SEMA7A* and *PLCD3*. The pairwise relationships for the genes in the associated loci are illustrated in Supplementary Fig. 5. In particular, this figure highlights that genes involved in embryonic development, such as *ROBO1*, *NRP2*, *GBX2*, *FGF3*, *PARD3B*, *SEMA7A* and *SHROOM3*, are closely connected. Also, *ROBO1*, *NRP2*, *GBX2*, *FGF3* and *SEMA7A* are involved in signalling pathways that regulate the migration of NCCs.

**Figure 2:**
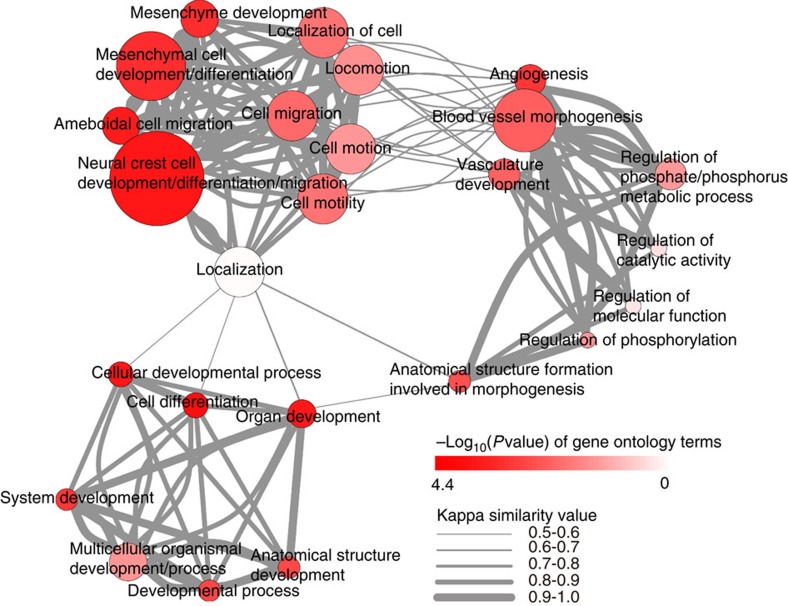
Graphic display of the similarity among the 25 gene ontology terms and their *P* values. The *P* value (Fisher's exact test) of each term and kappa similarity among terms were derived from the Database for Annotation, Visualization and Integrated Discovery (DAVID). The 25 nodes represents 25 gene ontology terms. The *P* values of the 25 nodes are indicated by the gradations in the colour red. The similarities between them are indicated by edges scaled according to their correlation (only correlations with a Kappa >0.5 are shown; the correlation are divided into 5 levels equally from 0.5 to 1) and node size represents connection times among nodes.

To investigate the enrichment of functional annotation, we used Database for Annotation, Visualization and Integrated Discovery (DAVID)^15^ to examine the 46 genes from the GRAIL analysis. The functional annotation clustering results are provided in Supplementary Data 7. Four DAVID-defined clusters displayed significant enrichment scores (ES) (Fisher's exact test *P*<0.05): (1) organ and system development; (2) cell differentiation, migration and development, especially for NCCs and mesenchymal cell; (3) vasculature development; and (4) regulation of phosphorus metabolic process. We used pairwise kappa similarity between terms from these four clusters to show their network structures (Fig. 2). We found the four clusters of biological processes correlated with each other. Many terms within the four clusters are relevant to the progresses of embryonic development, noting that the differentiation and migration of NCCs and mesenchymal cells play paramount roles in craniofacial morphogenesis.

To further explore the CFM candidate genes and their expression patterns, we analysed the 13 associated loci with the Data-Driven Expression-Prioritized Integration for Complex Traits (DEPICT) tool^16^. This analysis showed that 11 significantly prioritized genes (*SHROOM3, DCAKD, NID2, PARD3B, ROBO1, ARID3B, KLF12, FGF3, EPAS1, EDNRB, FRMD4A*; with a false discovery rate<5%) had functional connections (Supplementary Data 8). Gene set enrichment analyses identified the enriched categories of ‘positive regulation of cell differentiation,' ‘abnormal neural tube morphology,' and ‘failure of initiation of embryo turning' from those genes. Tests of enrichment of expression in particular tissues and cell types further identified 25 significant categories (*t*-test *P*-value<0.05), including 3 entries from the cardiovascular system, 6 from the musculoskeletal system, 6 from stem cells and 4 from the connective tissue cells (Supplementary Fig. 6). These significant categories were closely related to each other and critical for embryonic development.

### Gene expression patterns in embryos and gene-editing mice

To investigate the expression patterns of our candidate genes in embryos, we interrogated the *in situ* hybridization data from the gene expression database of the Mouse Genome Informatics, the Gallus Expression *in situ* Hybridization Analysis, and the Xenbase. All the candidate genes were expressed in the above database, and 10 of them were expressed in pharyngeal arches from where the craniofacial structures developed (Supplementary Fig. 7 and Data 9). It is notable that all the candidates expressed in the CFM-influenced organs during embryogenesis, such as cranial ganglion, mandible and sensory organs of ear and eye.

Considering that all studied CFM patients had external ear malformation, we measured the expression levels of the candidate genes of *ROBO1*, *EPAS1*, *KLF12*, *SHROOM3*, *NRP2*, *EDNRB*, *ARID3B*, *SEMA7A*, *PLCD3*, *FGF3* and *GBX2* by quantitative reverse transcription–PCR using the external ear tissues of BALB/c mice at 18 d.p.c. (days post coitum), 0 d.p.n. (days postnatal), 5 d.p.n. and adult. The results showed that mRNAs of *ROBO1*, *EPAS1*, *KLF12*, *SHROOM3*, *NRP2*, *SEMA7A* and *EDNRB* were detectable in the external ear of these four stages of mice development (Supplementary Fig. 8).

To understand the phenotypic consistency between CFM and mutant mouse models of the candidate genes, we interrogated the phenotypes of gene-editing mice deposited in the database of Mouse Genome Informatics. Mutant mice of nine candidate genes (*ROBO1*, *GATA3*, *GBX2*, *FGF3*, *NRP2*, *EDNRB*, *SHROOM3*, *SEMA7A* and *ARID3B*) were characterized by malformations of craniofacial system (Supplementary Fig. 7 and Data 10). Many mutant mice even shared similar phenotypes with CFM, such as abnormal craniofacial bone morphology, abnormal ear development and abnormal cranial ganglia morphology, which indicated the involvement of the candidate genes in the development of craniofacial structure.

### WGS on 21 CFM patients

To identify the potential causal mutations within the 13 associated loci, we performed WGS on 21 selected CFM patients from our study. We focused on novel mutations (not documented in dbSNP 138) of the following types: missense, frameshift, splice-donor and stop-gained. Finally, we obtained 40 missense and 2 frameshift mutations within 1-Mb genomic region surrounding the lead SNPs, and 20 of them had a PolyPhen score >0.6 (Supplementary Data 11). Supplementary Data 12 illustrates the potentially functional novel mutations in the CFM candidate genes. For 4 of the 21 patients, each had a novel missense mutation in *GATA3*, *SHROOM3*, *KLF12* and *PLCD3*, separately. Results from the functional analyses indicate that p.M2R in SHROOM3 and p.A20S in GATA3 are deleterious to the corresponding protein. p.M20V in KLF12 may modify the local secondary structure of the protein (Supplementary Fig. 9). p.R291H in PLCD3 may disrupt an H-bond between amino acids 291 and 286 (Supplementary Fig. 10), which may change the energy level (from −281.151 to −14.364) at 291 site and potentially lead to the instability of local structure.

## Discussion

Gestational exposure to teratogens supports the notion that the environmental factors contribute to CFM. However, various susceptibility loci identified from CFM or CFM-related syndromes indicate the critical involvement of genetics in this congenital disease^3^,^17^. Here we performed the first GWAS on CFM, identifying eight genome-wide significant loci and five implicated loci, which jointly explain 8.9% of the variance in susceptibility to this craniofacial anomaly. Several CFM-related genes or mutations have been proposed. Importantly, the candidate genes, except *FGF3* (ref. 18), within the 13 loci were newly reported in association with CFM. Our findings not only identify new risk loci for CFM, but also imply the complexity of genetic aetiology of this malformation.

Our results suggest that the candidate genes within the 13 associated loci are strongly correlated with the craniofacial development. First, the most prominent finding is that many of our candidate genes, such as *ROBO1*, *GBX2*, *NRP2*, *EDNRB* and *FGF19*, are functionally connected to each other and involved in NCCs and mesenchymal cells development and vasculogenesis. It is well known that craniofacial structures are derived from the first and second pharyngeal arches, which are composed of mesenchymal cells of cranial neural crest and mesodermal origin^4^. Second, the cell type-specific enrichment analyses based on the ENCODE or Roadmap projects indicate the enrichment of CFM-associated variants in regulatory elements of embryonic stem cells, which implies their potential roles in embryonic development. Third, *in situ* hybridization in the embryos of mouse, chicken and frog demonstrate that many of our candidate genes, such as *ROBO1*, *FGF3*, *EPAS1*, *KLF12*, *ARID3B*, *GBX2*, *EDNRB* and *NRP2*, are highly expressed in the pharyngeal arches and their derivatives of CFM-related craniofacial substructures, such as jaw, ear and eye. Fourth, mutant mice of the candidate genes frequently exhibit abnormalities at pharyngeal arches and the craniofacial region^19^,^20^,^21^,^22^,^23^,^24^,^25^. For example, *Ednrb* or *Arid3b* mutant mice have abnormal pharyngeal arch morphology^24^,^25^. Mouse embryos deficient in *GBX2* display aberrant migration and patterning of NCCs through disrupting the Slit/Robo signalling pathway^21^,^23^. Mutations in *SHROOM3* lead to cranial neural tube defects in mice^22^. Altogether, our findings reinforce the involvement of these genes in the pathogenesis of CFM.

NCCs are generated at the dorsal of the neural tube and subsequently undergo processes of delamination, transition, migration, patterning and differentiation into multiple cell types, which contribute to the formation of peripheral nervous system, craniofacial cartilage and bones and pigment cells^4^. Many of candidate genes identified in this study participated in all steps of NCCs development. *SHROOM3* plays a critical role in neural tube closure^26^. *GBX2* activates the expression of *ROBO1* involved in the Slit-Robo signalling that controls the motility and localization of NCCs^27^. *NRP2* is involved in the Sema-Nrp signalling, which shapes the NCCs migration streams by marking NC-free regions^28^. *SEMA7A* may also be involved in the Sema-Nrp signalling due to its widespread expression in cranial NCCs^29^. *EDNRB* is highly expressed in neural crest-derived head mesenchyme and determines the migration path of NCCs^30^. *ARID3B* and *FGF3* encode integrant for the identity, survival and differentiation of chondrogenic NCCs^31^,^32^, and the FGF signalling is also important for the homing process of NCCs^33^. In summary, many of the CFM candidate genes participate in the migration and differentiation of NCCs, and subsequently affect the formation of the NCCs-derived craniofacial organs.

NCC development disturbance has been well-accepted in the pathogenesis of CFM, while the hypothesis of vascular disruption is also noticeable^3^. Disruption in the development of the blood vascular system in an embryo can result in local ischaemia and birth defects^34^. In this study, *EPAS1* is found as a candidate gene for CFM. *EPAS1* is highly expressed in pharyngeal arches and vascular endothelial cells to regulate several genes involved in the development of blood vessels^35^. Meanwhile, one of the fates of NCCs is to differentiate into vascular endothelial cells and, later, to build up the vascular wall^4^. Although more studies are needed to reveal the relationship between *EPAS1* and NCCs, NCCs disturbance and vascular disruption may act synergistically to result in the facial malformation.

Our results significantly improve our understanding of the genetic pathogenesis of CFM. However, further studies are required to strengthen our findings. First, future GWAS and subsequent meta-analysis with world-wide CFM patients are expected to validate those associated loci, as well as to identify new ones. Second, deep-sequencing more DNA samples at the associated loci would help to identify the causative variants for CFM with next-generation sequencing technologies. Third, those associated variants mapped to regulatory elements require functional validation in their relevant cell types, such as NCCs and stem cell lines. Taken together, our study finds several new risk loci for CFM and connects the candidate genes to biological processes of NCCs migration and differentiation. The results not only highlight the genetic architecture of CFM, but also provide new clues for other craniofacial anomalies or syndromes.

## Methods

### Samples

We collected 1,382 congenital CFM patients from Plastic Surgery Hospital of Peking Union Medical College as a case cohort for a GWAS study. The cohort was composed of 1,056 males and 326 females with a mean age of 11.9 years old (s.d.: 6.5; range 4–48 years). Most of the patients were presented as a unilateral anomaly (1,256 individuals, 90.9%), with the right side being affected in nearly 61.7% (775 individuals). More details on phenotypes were illustrated in Supplementary Data 13. Among them, 1,308 patients had a record of geographic location and 71% of them were from northern China (using the boundary suggested by Xu *et al*.^36^). The control cohort was composed of 3,681 individuals with 2,362 males and 1,319 females, and was collected from several medical examination centres located in both northern and southern China. The percentage of control samples from northern China was 69.7%, not significantly (two-tailed *χ*^2^-test *P*=0.52) different from that of the case cohort.

All participants signed informed consent forms for biological investigations. This project was reviewed and approved by the Ethics Committee of the Plastic Surgery Hospital, Chinese Academy of Medical Sciences and Beijing Institute of Genomics, Chinese Academy of Sciences, in adherence with the Declaration of Helsinki Principles.

### Genotyping and quality control

All DNA samples were extracted using DNA-extraction kits (Tiangen Biotech). At the discovery stage, 942 cases and 2,020 controls were randomly loaded in 96-well plates and genotyped with the Human Omni-Zhonghua chips (Illumina) according to the manufacturer's specifications. Genotyping module of Genomestudio v3.0 (Illumina) was used to call the genotype of about 0.9 million SNPs. All DNA samples were successfully genotyped at a call rate >99.7% with a genotype call threshold (boundary for calling genotypes relative to its associated cluster) of 0.15. The genotype reoccurrence rate for three duplicated individuals was 99.99% on average.

To obtain high-quality data for GWAS, we pruned the data set of discovery stage with the following criteria: sample call rate >99%; SNP call rate >95%; and a threshold for Hardy–Weinberg equilibrium of 0.0001 (Fisher's exact test) in control cohort (Supplementary Fig. 11). In addition, to exclude closely related individuals, we calculated genome-wide identity by descent (IBD) for each pair of samples. We found that one pair of case and eight pairs of control have IBD >0.05, and removed one from each pair for the subsequent analyses. Due to limited power of rare variants in an association study, we only kept SNPs with minor allele frequencies >0.01. We extracted genotype data of the Yoruba in Ibadan (YRI), Utah Residents (CEPH) with Northern and Western European Ancestry (CEU), Japanese in Tokyo (JPT), Han Chinese in Bejing (CHB) and Southern Han Chinese (CHS) populations from the 1KG project and performed a principal component analysis (PCA) on these samples along with our genotyped samples using smartPCA package^37^. Asian populations (including CHB, CHS, JPT, and our samples) were clustered together, while Chinese samples were well separated from the Japanese samples (Supplementary Fig. 12). All Chinese samples were clustered into two subgroups, consistent with the notion of two different populations of northern and southern Chinese. We found that two outliers (based on genome-wide IBS) existed within our patients and were removed from subsequent analyses. In the end, we obtained 939 cases and 2,012 controls with 792,342 SNPs for our GWAS analyses. The total genotyping rate was 99.86%.

Genotyping for the lead SNPs in the 13 loci was done in additional 446 cases and 1,669 controls using the MassARRAY system from Sequenom. Three samples with more than 5% missing genotypes were removed from the data analysis. Fourteen SNPs had less than 5% missing genotypes and showed no deviation from Hardy–Weinberg equilibrium (*P*>0.05, Fisher's exact test) in control samples.

### Genetic power calculation

We used CaTS^38^ to estimate the statistical power of the current sample size. Under a multiplicative model, we set the case number at 942, control number at 2012, and a disease prevalence rate at <0.001, then estimated the power to obtain a significant level of 0.05, 1 × 10^−4^ and 5 × 10^−8^ at disease allele frequency (DAF) of 0.1, 0.05 and 0.01, respectively (Supplementary Fig. 13). Although the power was limited under the current sample size, we still had 80% chance to obtain genome-wide significant SNPs (*P*=5 × 10^−8^, the Bonferroni-corrected significance threshold) with genetic relative risk (GRR)=1.7 and a minor allele frequency=0.1, or GRR=2 and DAF=0.05, or GRR=4 and DAF=0.01.

### Association test

We estimated the associations between SNP genotypes and CFM traits by applying LRs in Plink (v1.9)^39^. To handle the population stratification of the samples, we performed LRs on all SNPs with a covariate of the first 20 eigenvectors from PCA. A QQ plot of this test was shown in Supplementary Fig. 14, of which the genomic inflation factor was 1.036 (based on median *χ*^2^). The Manhattan plots were constructed using qqman^40^. Bonferroni adjustment was corrected for multiple comparisons, and the threshold for genome-wide significance was set at a *P* value <6.3 × 10^−8^ (=0.05/792,342 variants). The regional association plots and linkage disequilibrium (LD) plots were performed using LocusZoom^41^. We performed more conditional LRs on the replicated samples with the first 20 eigenvectors from PCA as covariate and carried out combined analyses on the discovery and replication data, male versus female subgroups, left- versus right-side-affected subgroups using METAL^42^ with the parameters as follows: EFFECT, Beta; Weights in *P* value-Based Analysis, sample size; and heterogeneity, Cochran's *Q*-test.

### Genotype imputing

Pre-phasing haplotypes of each significantly associated locus was performed by SHAPEIT algorithm^43^. Imputing the untyped SNPs within a CFM-associated locus was based on the 1KG project phase 1 integrated variant set (b37; December 2013) with IMPUTE2 (ref. 44). In order to remove poorly imputed SNPs, we used a strict cutoff (info of 0.85) for post-imputation SNP filtering. LRs, controlling for the first 20 eigenvectors from PCA, were performed to test for the associations of imputed variants with CFM.

### Conditional association analysis

To identify other independently associated SNPs at a significant locus, we performed a conditional analysis on genotyped and imputed data using Plink. We first conducted association tests on the remaining significant SNPs by adjusting for the most significantly one at that locus. We then repeated the test with adjustment of the most significant one plus the remaining variants until no further genome-wide significant SNPs could be remained. Independently associated SNPs were those who have *P* value <0.05 after Bonferroni's adjustment in conditional association test.

### Joint multiple-SNP analysis for association study

To interrogate the interactions of SNPs within a gene or a defined haplotype block, we performed joint analyses. We defined 18,414 gene sets harbouring 358,890 SNPs and 120,458 block sets harbouring 606,013 SNPs. To test for joint effects with SKAT package^11^, multiple LR was implemented with the first five eigenvectors of PCA as covariates and with polyphen scores^45^ as each SNP's weight. The thresholds for adjustment of multiple tests were set at 2.72 × 10^−6^ (0.05/18,414 sets) and 4.15 × 10^−8^ (0.05/120,458 sets) for gene set and haplotype-set-based regressions, respectively.

### Functional annotation

We annotated the CFM-associated variants (typed and imputed) using SeattleSeq (v138) and HaploReg (v2)^12^. For SeattleSeq, we just kept variants that might have functional effects (Supplementary Data 2). For HaploReg, we only queried variants with a GWAS *P* value <1 × 10^−4^. All the annotations were displayed in Supplementary Data 3. The LD calculation was based on the ASN populations from 1KG (phase 1), and LD threshold (*r*^2^) was set at 1.0. The enrichment analyses of enhancer and DNase hypertension site were performed based on the ENCODE and the Roadmap databases with 1KG ANS pilot data as background set.

### eQTL analysis

To interrogate the associated SNPs with regard to gene expression, we performed eQTL analyses on SNPs with a GWAS *P* value <0.01 using Genevar (v3.3.0) a platform of database and web services, designed for data integration, analysis and the visualization of SNP–gene associations^13^. With a SNP-centric approach, we used SNP–gene association analyses with genetic variations and gene expression profiling data from lymphoblastoid cell lines of the CEU, CHB, GIH, JPT, LWK, MEX, MKK and YRI individuals from HapMap. We measured the effects with the parameters set to Spearman's rank correlation coefficients and with a window size of 200-kb and a *P* value threshold of 0.01. For the seven HapMap populations, significant eQTL SNPs associated with gene expression are illustrated in Supplementary Data 4.

### Estimation of CFM variance explained

We used the GCTA package^46^ to estimate the variance in CFM liability, which could be explained by either the associated SNPs or all genotyped SNPs. The prevalence of CFM was 1.4 per 10,000, estimated from a 5-year epidemiological study in China^10^. For each associated locus, we used a SNP set composed of SNPs with a *P* value <0.05 in that locus to estimate the phenotypic variance that could be explained.

### Candidate gene prediction and pathway analyses

We used GRAIL^14^ to analyse the potential relationships of the residing genes in the 13 associated loci without phenotype information. The query regions comprised the 200-kb flanking regions of a lead SNP (if no gene was found, then the nearest gene to that SNP was picked). The analysis settings were as the following: human genome assembly, HG18; HapMap population, CHB+JPT; functional data source, PubMed Text (August 2014); gene size correction, off; gene list, default gene list; queries and seed regions, equal.

To perform gene-annotation enrichment analyses and functional annotation clustering, we analysed the 46 genes from GRAIL using the DAVID v6.7 (ref. 15). Modified Fisher's exact test was used to determine the significance of gene-term enrichment. The ES was used to rank the overall enrichment of the annotation groups. The ES value was defined as minus log transformation on the average *P* values of annotation terms and was set at 1.3 (non-log scale of 0.05) for significance. To trim the annotation clusters, we used high-classification stringency parameters set suggested by DAVID. To depict the relationship among gene ontology terms within a significant cluster, we used R language to illustrate the kappa similarity between the terms.

We also used DEPICT to systematically identify the most likely causal genes in a CFM-associated locus with regard to the highly expressed tissues and cell typed and enriched physiological condition^47^. We first retrieved independent 13 sets of loci using clump methods in Plink (parameters of --clump-p1 1e-5 --clump-kb 500 --clump-r2 0.1). We then submitted them to DEPICT and obtained 13 non-overlapping genomic regions (similar to our previous identified 13 loci) with a total of 29 genes. Meanwhile, gene expression level and physiological system enrichment were also analysed using various databases of gene expression, protein–protein interactions, Mouse Genetics Initiative, Gene Ontology and pathways of Reactome and KEGG.

### Gene expression in embryos and gene-editing mice

To investigate the expression pattern of the candidate genes in embryos, we interrogated *in situ* hybridization data of *ROBO1*, *GATA3*, *GBX2*, *FGF3*, *NRP2*, *EDNRB*, *SHROOM3*, *SEMA7A*, *EPAS1*, *KLF12*, *PLCD3* and *ARID3B* using the database of Mouse Genome Informatics, Gallus Expression *in situ* Hybridization Analysis and Xenbase. We focused on the NCCs-related tissues and CFM-influenced facial substructures. To explore the phenotypes of mutant mice caused by these candidate genes, we interrogated the database of Mouse Genome Informatics and focused on embryonic substructures that related to the craniofacial development.

External ear's malformation was a common character to CFM. The development of external ears is completed at 5 d.p.n. for mouse. We collected the external ears from BALB/c lineage at 18 d.p.c. (3 samples, 1 male and 2 females), 0 d.p.n. (3 samples, 2 males and 1 female), 5 d.p.n. (3 samples, 2 male and 1 females) and adult (4 samples, 3 males and 1 females), respectively. Frozen tissues were disrupted and homogenized in RLT Buffer. Total RNA was extracted from the ear tissue samples with the traditional TRIzol method, quantified with a Nanodrop spectrophotometer (Thermo Fisher Scientific). The quality of RNA was confirmed with agarose electrophoresis. The total RNA was reverse transcribed into complementary DNA (cDNA) in a 20-μl reaction using a FastQuant RT Kit (YQYK-biotech). For quantitative reverse transcription–PCR amplifications, gene-specific primers for *ROBO1*, *ARID3B*, *SEMA7A*, *FGF3*, *FGF4*, *EPAS1*, *KLF12*, *GBX2*, *SHROOM3*, *NRP2*, *EDNRB* and *PLCD3* were from Sangon-biotech (Supplementary Table 3). A genomic quantitative real-time PCR was performed with the 7500 Real-Time PCR system (Applied Biosystems). In a 10 μl PCR reaction, 5 μl of SYBR Green Master mix (Applied Biosystems), 30 ng of cDNA, and 10 pmol of each primer were included. The expression level of *GAPDH* was measured in parallel as an internal control for normalization. Amplification efficiency was confirmed by melt curve analysis demonstrating the absence of nonspecific products or primer-dimers. Three replicates were performed for each biological sample at the reverse transcription step and the same batch of cDNA was used for all subsequent PCR amplifications. The relative expression level was determined using the 2^−ΔCt^ method^48^. This experiments was reviewed and approved by the Ethics Committee of the Plastic Surgery Hospital, Chinese Academy of Medical Sciences, in adherence with the Declaration of Helsinki Principles.

### Whole-genome sequencing

We sequenced the whole genome of 21 CFM patients from our study samples, including 7 left-side-affected, 7 right-side-affected and 7 bilateral individuals. The selected individuals were those who had risk alleles (with a frequency greater in cases than in controls) of the lead SNPs rs17802111, rs3754648, rs13089920, rs10905359, rs11263613 and rs10459648 for right-side-affected CFM, rs13089920, and rs17090300 for left-side-affected CFM, and rs13089920 for bilateral CFM. Paired-end sequencing with 150-bp read lengths was performed on Illumina HiSeq X10 instrument and yielded a mean depth of 27 ×. All reads were mapped to the human reference genome (hg19) using BWA^49^ (version 0.7.5a). PCR duplicates were removed using the Picard software program (version 1.92; http://broadinstitute.github.io/picard/). The Samtools^50^ (version 0.1.19) and GATK^51^ (version 3.1) software packages were used to call variants. Within the 13 associated loci, we annotated variants with SeattleSeq Annotation 138 and removed variants that had been reported in dbSNP 138. Then we focused on the missense, frameshift, splicing and conserved (GERP score >2 or phastCons score >0.8) variants, as well as variants in TFBSs. All variants in Supplementary Data 11 pass manual confirmation using the IGV package^52^.

### Functional analyses on variants

We performed functional analyses on all identified candidate variants with following steps. First, we evaluated possible impacts of the mutations on the structures or functions of the corresponding proteins using Polyphen-2 (ref. 53) and SIFT^54^. Mutations with PloyPhen score >0.5, or SIFT score <0.05 were considered as deleterious to the function or structure of protein. Second, SignalP 4.1 was used to predict the signal peptide with the assumption that the protein contained no transmembrane segments^55^. The parameters for analysis with SignalP were as follows: Organism group, Eukaryotes; D-cutoff values (optimize the performance and affect sensitivity), Default; Method, Input sequences do not include transmembrane segments. Third, we predicted the secondary structure of both the wild-type and mutant proteins using an online software PSIPRED (v3.3)^56^. Fourth, we used SWESS-MODEL to predict the tertiary structure of each protein and found that mutations were not in any range of modelled residues except p.R291H in PLCD3. We searched the three-dimensional (3D) structure deposited in the Research Collaboratory for Structural Bioinformatics Protein Data Bank (RCSB PDB). We found that only GATA3 had X-ray-derived 3D structure, but p.A20S in GATA3 was not in the fragment of unknown structure. Fifth, based on modelled 3D structure of PLCD3, we used Swiss-PdbViewer 4.1 (ref. 57) to view the effect of p.R291H on the protein PLCD3. We downloaded Q8N3E9-PLCD3 protein from SWISS-MODEL repository and analysed the wild-type and mutant proteins using parameters as follows: minimum energy, residues within six angstroms to the p.R291H, secondary structure as ribbon format, colourful secondary structure by types, computing H-bonds and van der Waals.

## Additional information

**Accession codes:** The craniofacial microsomia chips data have been deposited in GEO under the accession codes GSE69664. The craniofacial microsomia WGS data have been deposited in SRA under the accession codes SRP067380.

**How to cite this article:** Zhang, Y.-B. *et al*. Genome-wide association study identifies multiple susceptibility loci for craniofacial microsomia. *Nat. Commun.* 7:10605 doi: 10.1038/ncomms10605 (2016).

## Supplementary Material

Supplementary Information Supplementary Figures 1-14 and Supplementary Reference

Supplementary Data 1 Associations of craniofacial microsomia risk with genotyped and imputed variants that reach significance level of P < 1 × 10^−4^.

Supplementary Data 2 Functional annotation of genotyped and imputed SNPs having P < 0.05.

Supplementary Data 3 Regulatory elements prediction and enrichment analysis on SNPs with GWAS P value less than 1 × 10^−4^.

Supplementary Data 4 SNP-centric eQTL analyses performed with lead SNPs and their close proxies (r^2^ > 0.8).

Supplementary Data 5 Regulatory elements prediction and enrichment analysis on cis-eQTLs.

Supplementary Data 6 Summary of GRAIL results for significantly and suggestively associated SNPs.

Supplementary Data 7 Functional annotation clustering on 46 genes within 13 associated loci.

Supplementary Data 8 Summary of DEPICT results.

Supplementary Data 9 The gene expression pattern in substructure of mouse at embryo or adult stage.

Supplementary Data 10 The phenotype of mutant mice

Supplementary Data 11 Novel(not reported in dbSNP 138) missense and frameshift variants identified in 21 craniofacial microsomia patients in 13 associated loci (1 Mb surrounding lead SNP) using whole genome sequencing method.

Supplementary Data 12 Potientially functional variants identified in 21 craniofacial microsomia patients in 13 associated loci using whole genome sequencing method.

Supplementary Data 13 The phenotypes of 1382 craniofacial microsomia patients in this study.

## Figures and Tables

**Table 1:** Most significantly associated risk variants with craniofacial microsomia.

Chr. (band)	Lead SNPs	Position	Alleles	GWAS (939 versus 2,012)*		Replication (443 versus 1,669)*		Combined (1,382 versus 3,681)*		
				*P*	OR (95% CI)	*P*	OR (95% CI)	Freq.	*P*	OR (95% CI)
*Genome-wide significant loci for all cases*										
2p21	rs17802111	46509657	A/G	1.44 × 10^−11^	1.48 (1.32–1.66)	5.45 × 10^−7^	1.47 (1.26–1.71)	0.46	9.57 × 10^−18^	1.48 (1.35–1.62)
2q33.3	rs7420812	206435709	G/A	2.01 × 10^−6^	1.33 (1.18–1.49 )	1.32 × 10^−4^	1.35 (1.16–1.58)	0.37	6.74 × 10^−10^	1.33 (1.22–1.46)
2q37.2	rs3754648	237021346	A/G	6.33 × 10^−9^	1.41 (1.26–1.58)	2.53 × 10^−5^	1.38 (1.19–1.61)	0.42	5.09 × 10^−13^	1.39 (1.27–1.53)
3p12.3	rs13089920	78552232	G/A	1.06 × 10^−70^	5.21 (4.34–6.25)	2.62 × 10^−46^	5.05 (4.05–6.31)	0.25	2.15 × 10^−120^	5.18 (4.51–5.95)
10p14	rs10905359	8449891	A/C	6.58 × 10^−9^	0.71 (0.63–0.80)	2.98 × 10^−3^	0.79 (0.68–0.92)	0.33	5.11 × 10^−9^	0.76 (0.69–0.83)
11q13.3	rs11263613	69661334	A/G	7.91 × 10^−12^	1.71 (1.47–2.00)	5.44 × 10^−6^	1.60 (1.31–1.96)	0.19	3.61 × 10^−17^	1.68 (1.49–1.89)
15q24.1	rs10459648	74865440	A/G	2.86 × 10^−12^	0.67 (0.59–0.75)	1.12 × 10^−10^	0.60 (0.52–0.70)	0.40	1.05 × 10^−23^	0.63 (0.58–0.69)
										
*Genome-wide significant locus for left-side-affected cases (case number for GWAS, replication, and combined set was 330, 151 and 481, respectively.)*										
13q22.1	rs17090300	74157451	A/G	6.26 × 10^−9^	2.42 (1.80–3.26)	1.36 × 10^−3^	2.04 (1.32–3.15)		1.04 × 10^−11^	2.31 (1.81–2.93)
										
*Suggestive significant loci with all cases*										
4q21.1	rs3923380	77468594	C/A	1.05 × 10^−6^	0.75 (0.67–0.84 )	5.32 × 10^−2^	0.86 (0.74–1.00)	0.38	8.19 × 10^−8^	0.78 (0.71–0.86)
10p13	rs4750407	13795471	G/A	3.02 × 10^−6^	1.32 (1.18–1.49)	2.69 × 10^−2^	1.19 (1.02–1.39)	0.36	2.49 × 10^−7^	1.27 (1.16–1.40)
13q22.3	rs9574113	78418131	G/A	4.12 × 10^−6^	0.73 (0.64–0.84 )	8.62 × 10^−2^	0.86 (0.72–1.02)	0.22	8.16 × 10^−6^	0.79 (0.71–0.88)
14q22.1	rs754423	52527187	G/A	4.14 × 10^−7^	1.32 (1.18–1.47 )	2.32 × 10^−2^	1.19 (1.02–1.37)	0.53	1.19 × 10^−7^	1.27 (1.16–1.38)
17q21.31	rs7222240	43136195	A/G	6.13 × 10^−6^	2.01 (1.48–2.75 )	1.05 × 10^−1^	1.39 (0.93–2.08)	0.04	9.49 × 10^−6^	1.73 (1.36–2.20)

Var_expl_ (%)	Het *P*value	Nearby genes	Candidate genes		
			GRAIL	DEPICT	NCC related
0.7849	0.6134	*PRKCE, EPAS1, TCONS_00004241*	*EPAS1*	*EPAS1*	*EPAS1*
0.4283	0.7895	*FARD3B, NRP2*	*PARD3B*	*PARD3B*	*NRP2*
0.7219	0.5576	*ASB18, AGAP1, GBX2*	*GBX2*	*—*	*GBX2*
2.1512	0.3708	*ROBO1*	*ROBO1*	*ROBO1*	*ROBO1*
0.6334	0.2967	*LINC00708, GATA3, GATA3-AS*	*GATA3*	*—*	*GATA3*
0.9065	0.2718	*CCND1, FGF19, ORAOV1, FGF3, FGF4, TCONS_00019371*	*FGF3*	*FGF3*	*FGF3*
0.3886	0.9419	*SEMA7A, UBL7, ARID3B, CLK3, EDC3, CCDC33, CYP11A1, TCONS_00023466*	*SEMA7A*	*ARID3B*	*ARID3B, SEMA7A*
0.9065	0.13	*KLF12, TCONS_00021669, TCONS_00021670, TCONS_I2_00006907, TCONS_I2_00007425*	*KLF12*	*KLF12*	*KLF12*
0.3657	0.07134	*FLJ25770, SHROOM3*	*SHROOM3*	*SHROOM3*	*SHROOM3*
0.6429	0.1577	*FRMD4A, PRPF18*	*FRMD4A*	*FRMD4A*	*—*
0.4057	0.1425	*SLAIN1, EDNRB, SCEL, LINC00446, LINC01069*	*EDNRB*	*EDNRB*	*EDNRB*
0.3904	0.1554	*NID2, GNG2, TCONS_00022504, TCONS_00022745,C14orf166*	*C14orf166*	*NID2*	*—*
0.158	0.09706	*ACBD4, CCDC103, KIF18B, PLCD3, HEXIM1, DCAKD, GFAP, FMNL1, HEXIM2, NMT1, C1QL1, C17orf46, EFTUD2*	*PLCD3*	*DCAKD*	*PLCD3*

Chr. (band), cytogenetic band; CI, confidence interval; DEPICT, candidate genes predicted by DEPICT; Freq., frequency of the effect allele in cases; nearby genes, genes (including LincRNAs (large intergenic non-coding RNAs)) spanning or flanking (<200-kb away from) the lead SNP from UCSC genome browser; GRAIL, candidate genes predicted by GRAIL; GWAS, genome-wide association study; NCC; neural crest cell; OR, odds ratio for the minor allele; Position, physical position of human genome version of hg19; SNP, (single-nucloetide polymorphism) rsID of the lead variant; Varexpl, variance in liability to microtia explained by the locus at the prevalence rate of 1.4/10,000 in China.

^*^The former is for cases number and the latter is for controls number.
