# Supplementary material for: Genome-wide association study identifies multiple susceptibility loci for craniofacial microsomia
Source: Nat Commun. 2016 Feb 8;7:10605. doi: 10.1038/ncomms10605 (PMC4748111; doi:10.1038/ncomms10605)
Supplement: Supplementary Information — Supplementary Figures 1-14 and Supplementary Reference [file ncomms10605-s1.pdf]

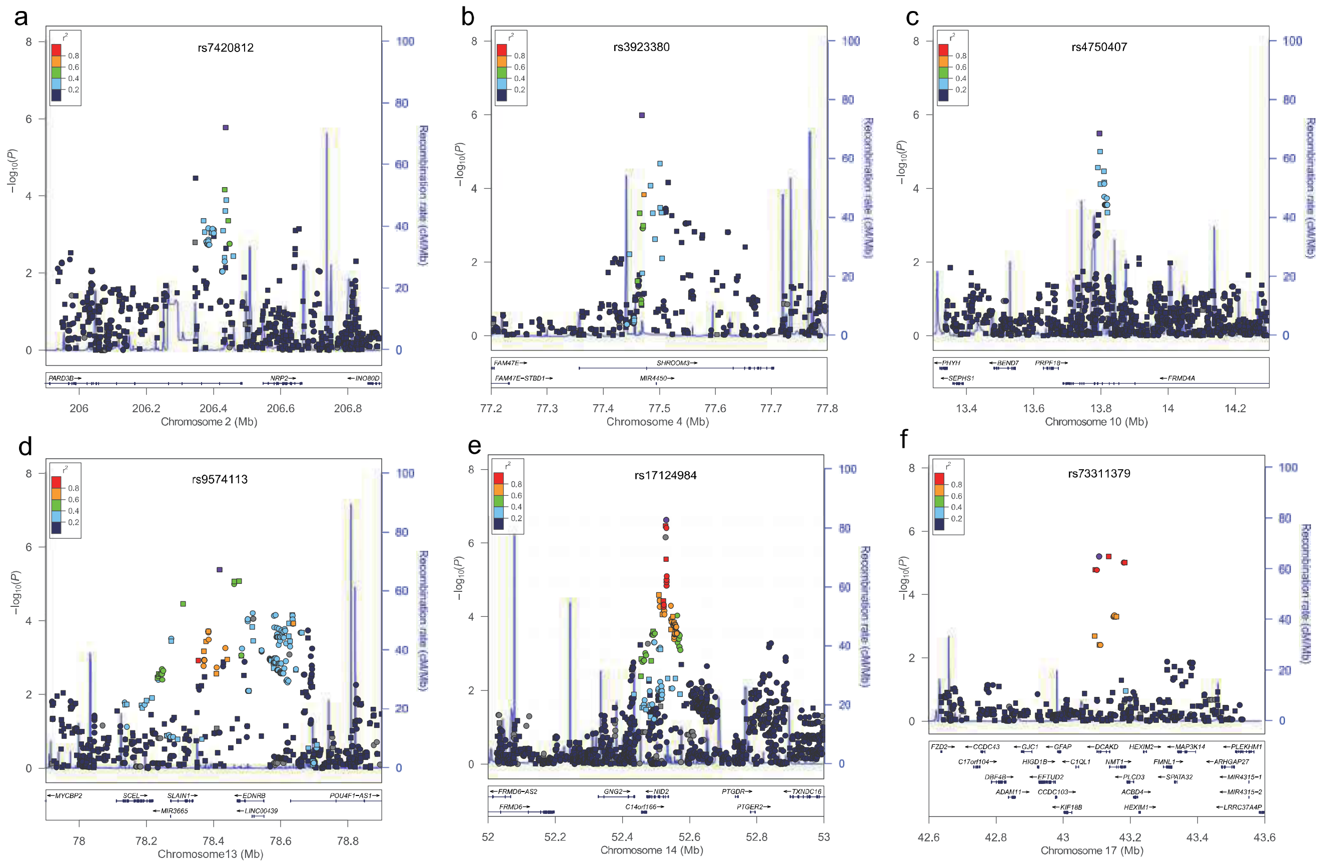

Supplementary Fig.1 Regional plots for the 6 suggestive significant loci at discovery stage. (a-g) The association of craniofacial microsomia risk with suggestive significant loci of 2q33.3 (a), 4q21.1 (b), 10p13 (c), 13q22.3 (d), 14q22.1 (e), and 17q31.31 (f). Each point represents an SNP plotted with its $-\log_{10}P$-value as a function of genomic position (hg19). Imputation analysis is shown with circles and direct genotyping is shown with squares. In each regional plot, the purple symbol denotes the lead SNP. The color coding of the rest of SNPs shows LD with the lead SNP: red , $r^2 \geq 0.8$; gold, $0.6 \leq r^2 < 0.8$; green, $0.4 \leq r^2 < 0.6$; cyan, $0.2 \leq r^2 < 0.4$; blue, $r^2 < 0.2$; gray, r^2 unknown. Recombination rates are estimated from ASN population of 1KG project (Mar 2012). Gene annotations are taken from the UCSC genome browser.

a

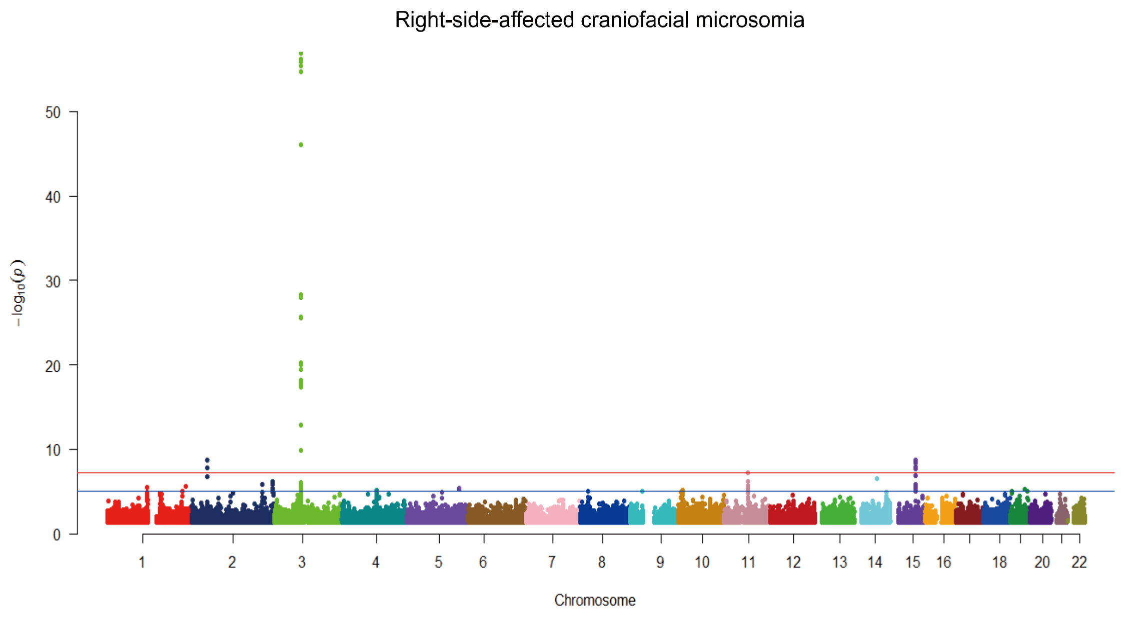

b

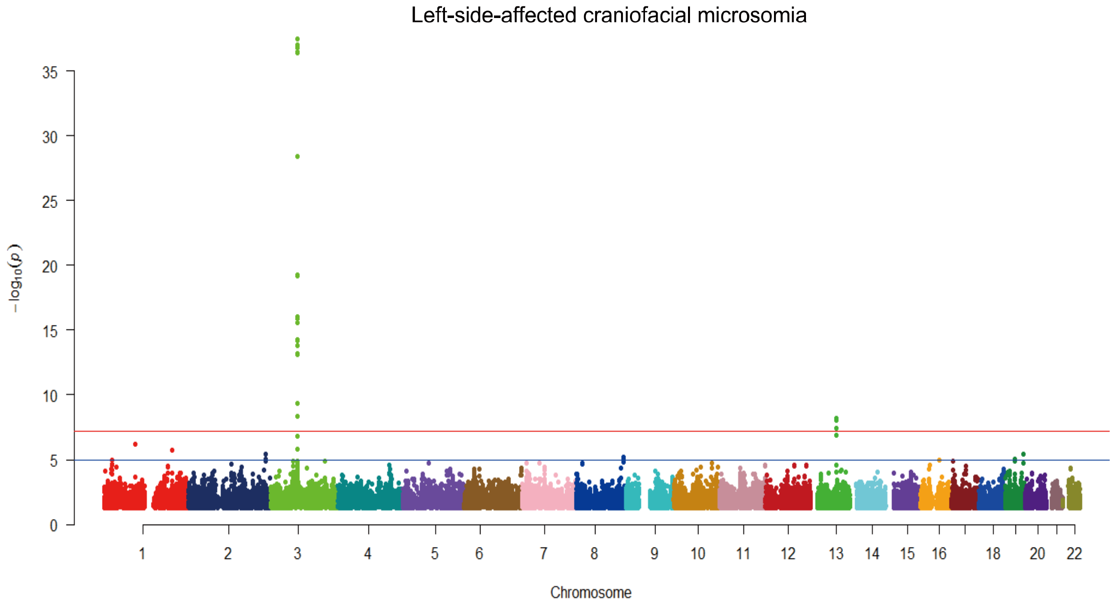

Supplementary Fig.2 Logistic regression analysis on left- and right-side-affected craniofacial microsomia. (a) Associations between genetic variants and right-side-affected craniofacial microsomia risk. For seven significant loci with all craniofacial microsomia samples, four (2p21, 3p12.3, 11q13.3, and 15q24) of them still showed genome-wide significant association with craniofacial microsomia, and two (2q37.2, 10p14) of them reached suggestive significance. (b) Associations between genetics variants and left-side-affected craniofacial microsomia risk. Only 3p12.3 is still significant in left-side-affected craniofacial microsomia, but 13q22.1 is a newly identified one for the subgroup. The $-\log_{10}$(*P*-value) of each SNP is shown as a function of genomic position on the autosome (hg19). Genome-wide significance (solid red line; $P \leq 6.3 \times 10^{-8}$) and suggestive significance (solid blue line; $P \leq 1 \times 10^{-5}$) are denoted. The samples size for the left- and right-side-affected CFM is 330 and 609, respectively.

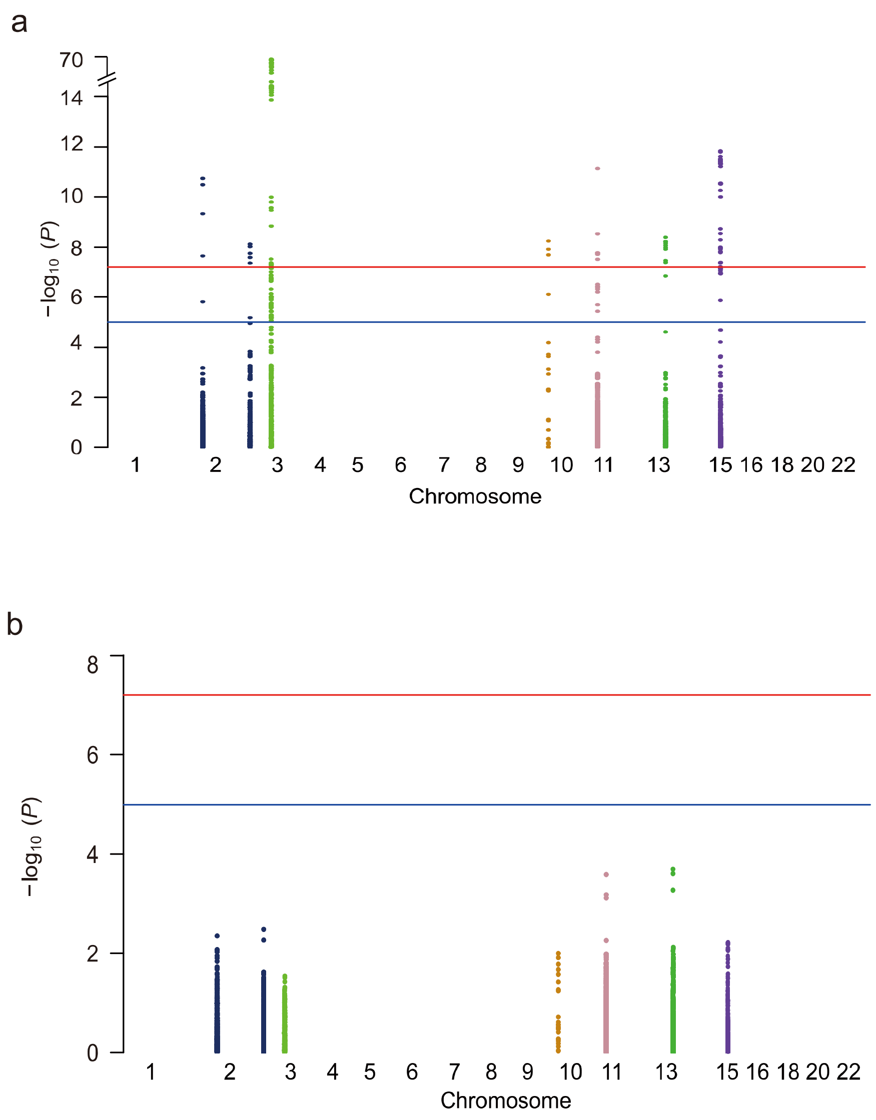

Supplementary Fig. 3 Imputation and conditional analysis on 7 genome-wide significant associated loci of CFM. (a) Imputation results of significantly associated loci with craniofacial microsomia. (b) Associations at each genome-wide significant susceptibility locus after conditional analysis with the genotype of the most significantly associated SNP at the locus. The $-\log_{10}$(P-value) of each SNP is shown as a function of genomic position on the autosomes (hg19). Genome-wide significance (solid red line; $P \leq 6.3 \times 10^{-8}$) and suggestive significance (solid blue line; $P \leq 1 \times 10^{-5}$) are denoted.

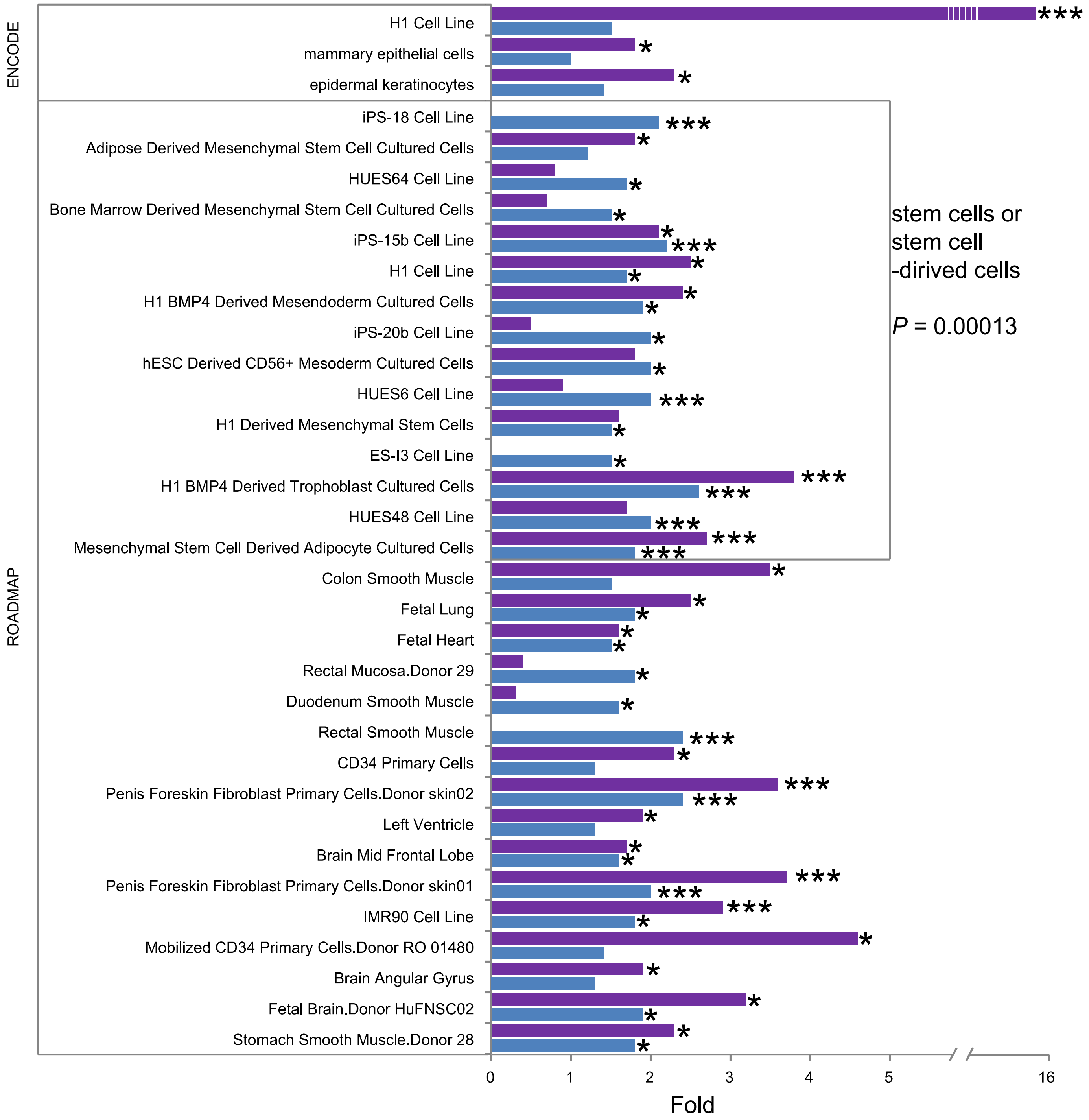

Supplementary Fig.4 Enhancer enrichment analysis for the CFM-associated SNPs based on ENCODE and ROADMAP epigenomics. X-axis shows the fold change for the observed against the expected enhancers. Y-axis shows tissue or cellular types that are enriched by enhancers. Purple bars represent the "strongest enhancers" and blue for all enhancers. Asterisk at each bar represent the significant level for enhancer enrichment in ROADMAP database: ***, $P \leq 0.001$; *, $0.001 < P \leq 0.05$; no asterisk, $P > 0.05$. Chi-square test was used to calculate the significant level for a cluster of stem cells or stem cell-derived cells.

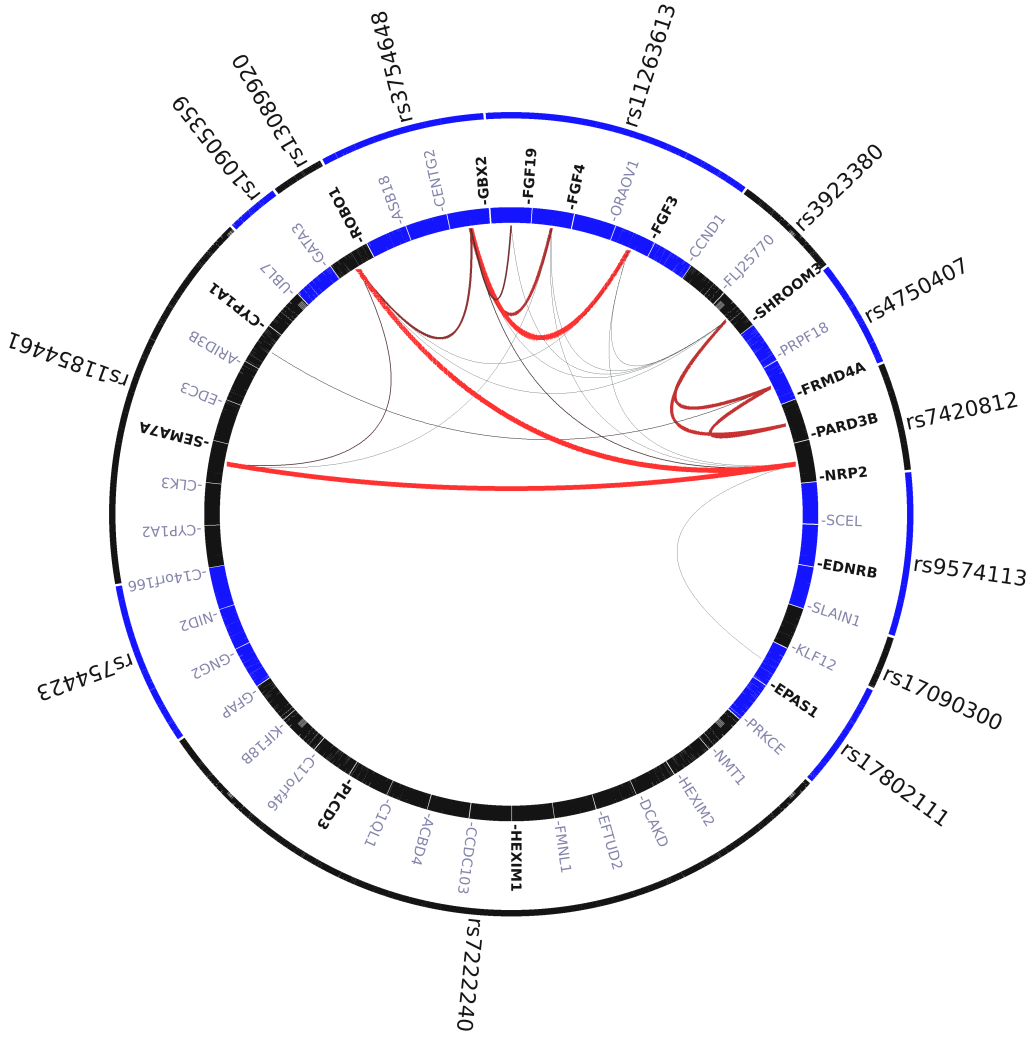

Supplementary Fig.5 Graphical display of the functional connection among the 13 associated loci. We submitted the 400-kb regions surrounding the lead SNPs of the 13 loci for GRAIL prediction. The outer circle shows the lead SNPs. The inner circle indicates the investigated genes with blue or black segments to represent the borderline between gene and loci. Black genes indicate connections from scientific publications; gray genes have no connections. Red and thicker lines represent stronger connections among genes.

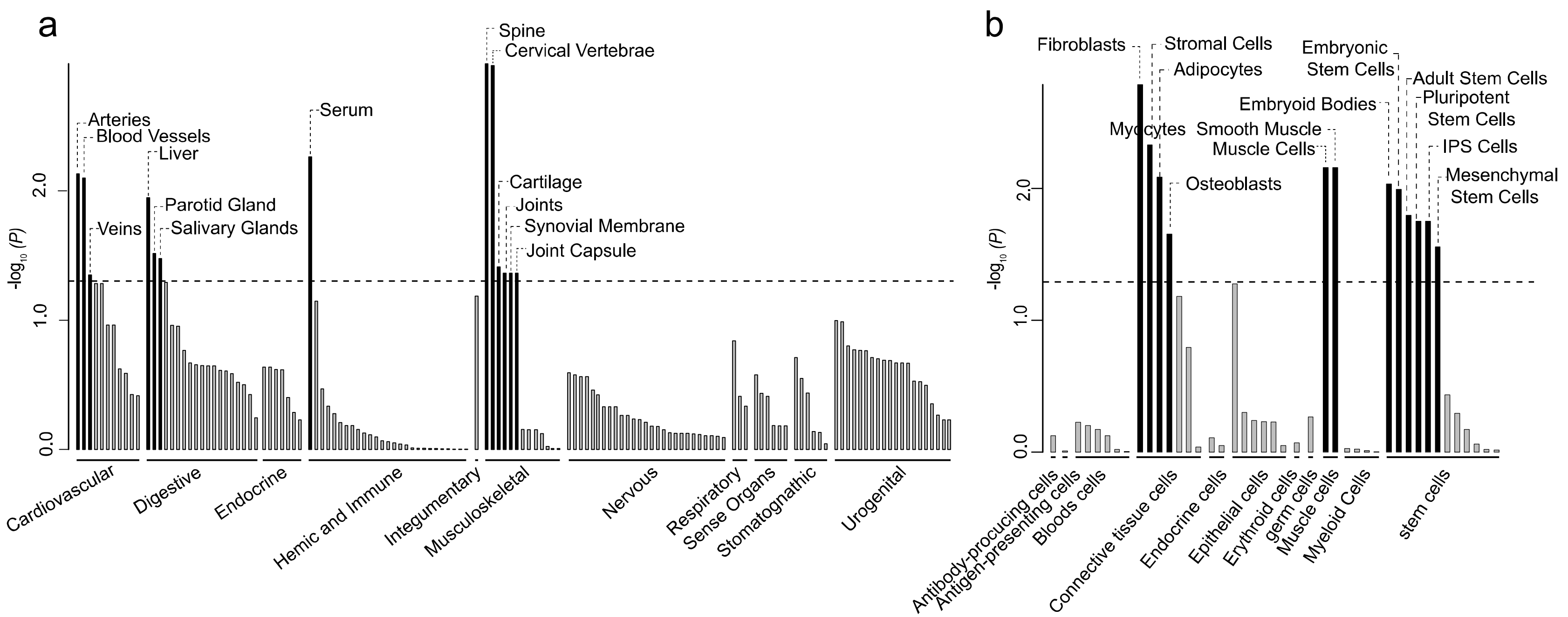

Supplementary Fig.6 Graphical display of the *P*-values in the physiological systems (a) and cell types (b). The terms with $P < 0.05$ are indicated by the solid bars and others are indicated by the unfilled bars.

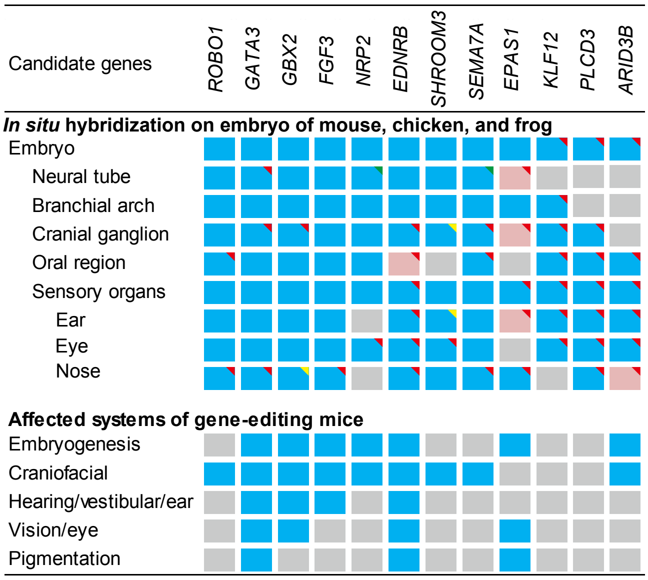

Supplement Fig.7 The expression pattern of candidate genes in embryos from animal models and from the gene-editing mice with CFM-related phenotypes. The gene expression profiles were from the database of Mouse Genome Informatics, Gallus Expression in Situ Hybridizaiton Analysis, and Xenbase. Rectangle with blue, pink, or gray color represents the mRNAs that were detectable, undetectable, or not recorded in the listed organ systems, respectively. Rectangle with red, blue, or yellow triangle mark represents expression data from mouse, chicken, or frog, respectively, for an item without a mark meant that a certain gene transcript was detected in all three animals. In chart of affected systems of gene-editing mice, a blue rectangle means that the abnormalities were observed, and a gray one means “not annotated”

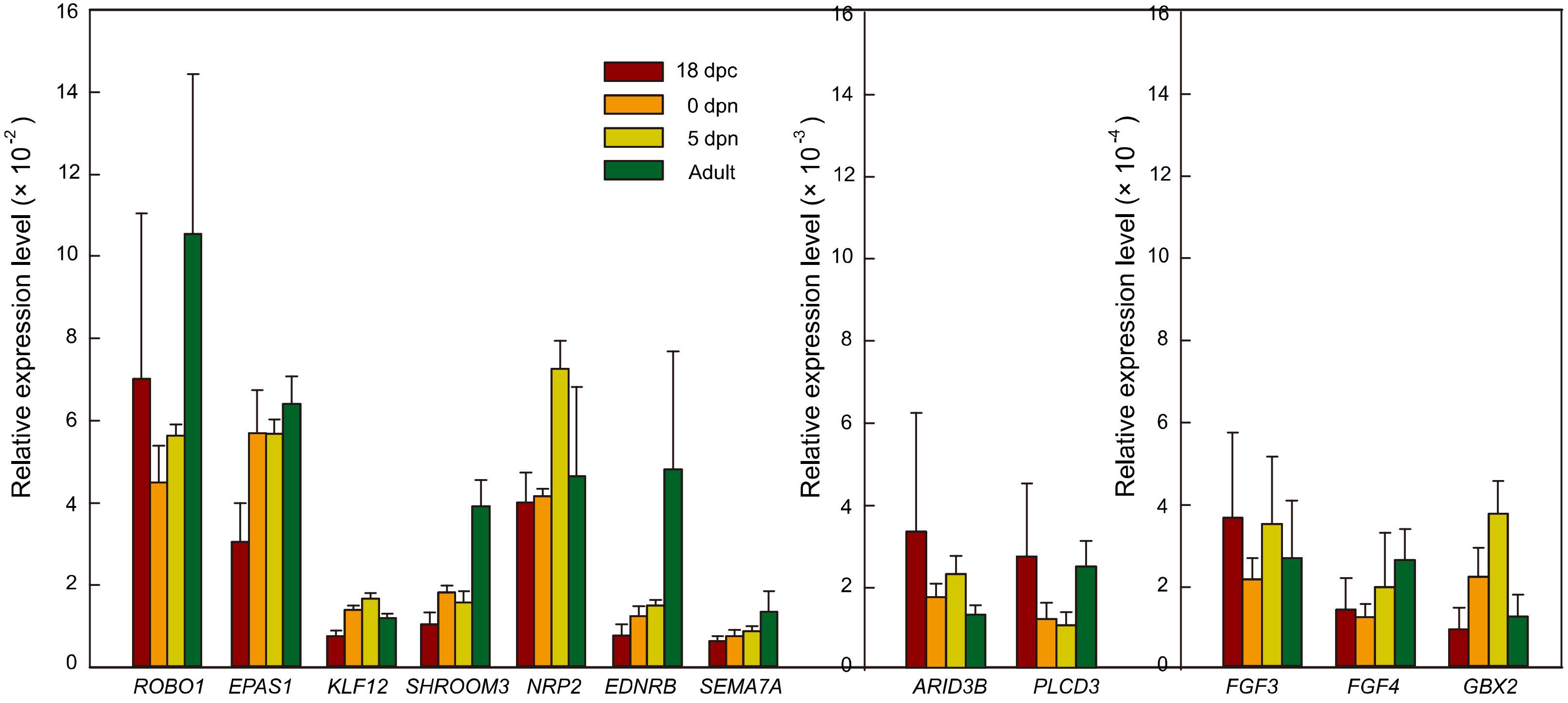

Supplementary Fig.8 Relative expression level of the candidate genes in the external ear of BLAB/c mouse lineage at 18 dpc (n =3), 0 dpn (n=3), 5 dpn (n=3), and adult (n=4). Each RT-PCR reaction was performed in triplicate. The relative expression level was determined using the $2^{-\Delta Ct}$ method.

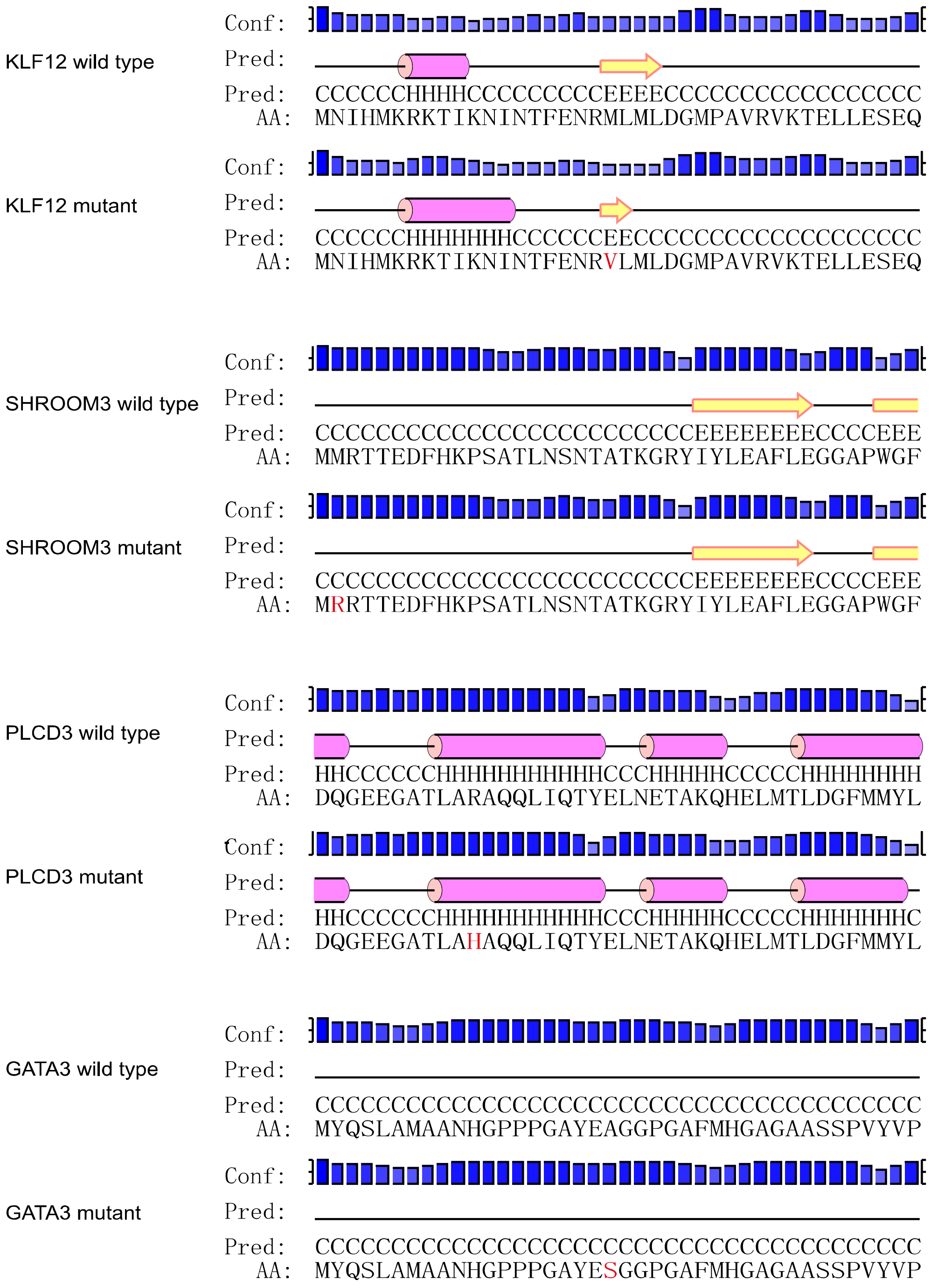

Supplementary Fig.9 The predicted secondary structure of the wild and mutant protein sequences flanking the mutations. The diagrams show the protein sequences with secondary structure and their confidence values at the aligned positions. The secondary structure is annotated as follows: alpha-helix, pink cylinder; beta-sheet, yellow arrow; coil, black line. Conf, confidence; Pred, predict; H in Pred line, Helix; C in Pred line, coil; E in Pred line, sheet; AA, amino acid; red letter in AA line, mutant amino acid.

a

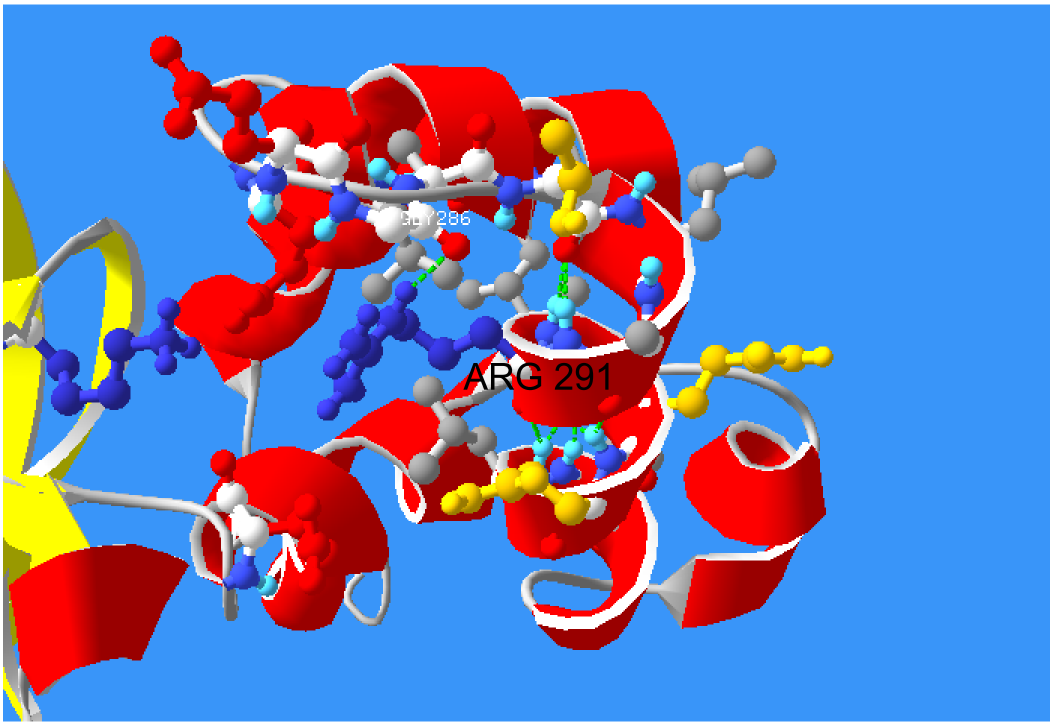

b

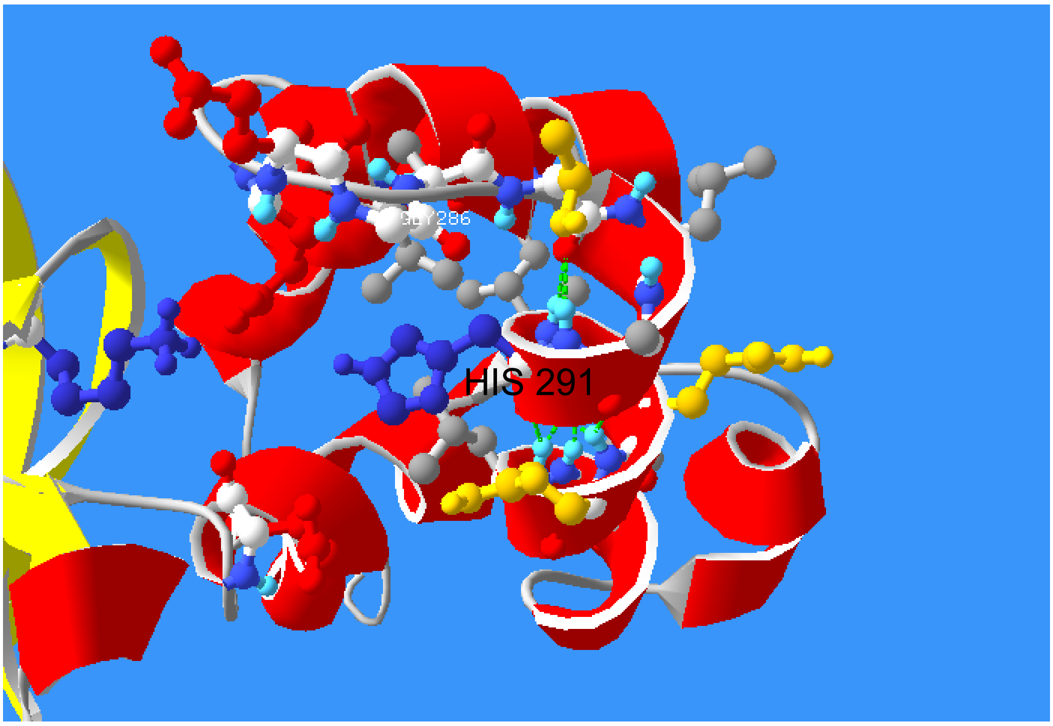

Supplementary Fig. 10 The partial tertiary structure of PLCD3 within 6 angstroms to the R291H. The wild type (A) and mutant (B) proteins were viewed with Swiss-PdbViewer. The alpha-helix is shown as red ribbon, beta-sheet as yellow ribbon, and coil as grey stick. The backbone of the chain is colored by CPK mode: white for carbon, red for oxygen, and blue for nitrogen. The sidechain of amino acids is colored as the following: Acidic, red; Basic, blue; Polar, yellow; and Non-Polar, gray. H-bonds is displayed as green dash line. The sidechain of HIS291 can’t form an H-bond with GLU286 for steric hindrance or cla shes in the mutant protein.

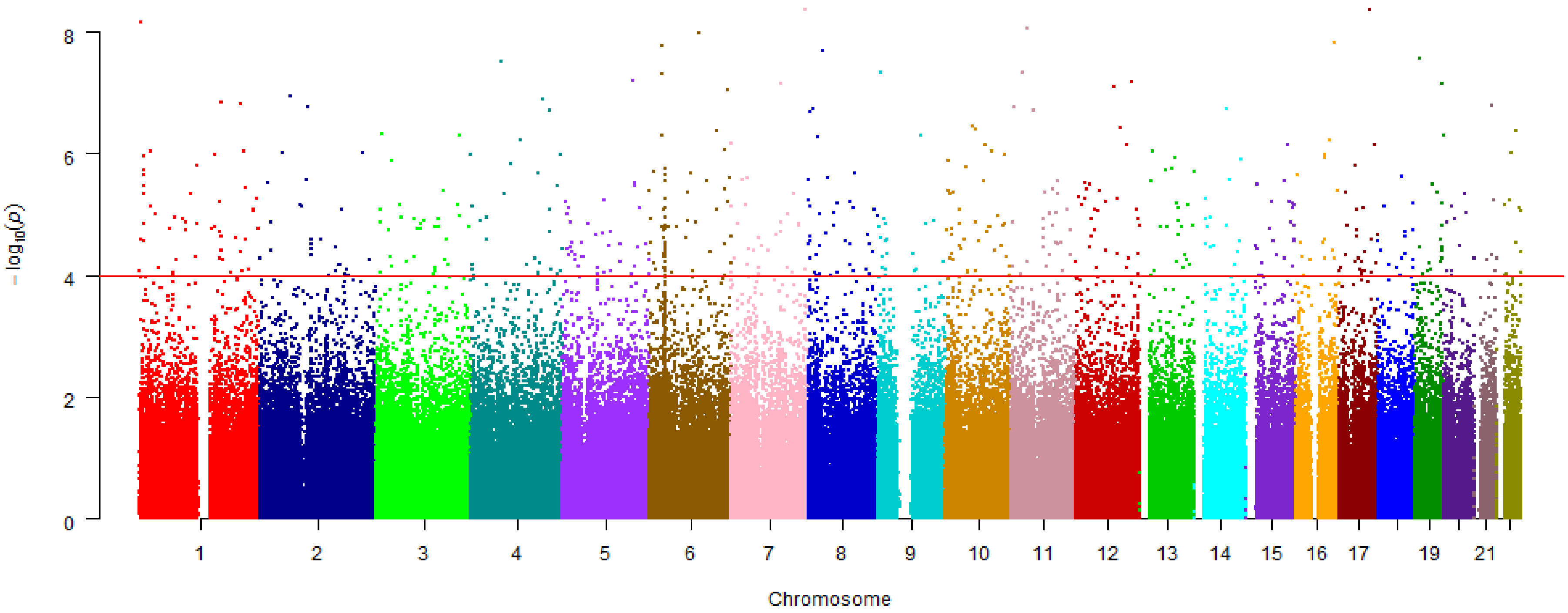

Supplementary Fig.11 Hardy-Weinberg equilibrium (HWE) tests on 2012 controls used in this study. X-axis shows SNPs along with chromosomes which are illustrated by different colors, and Y-axis shows the $-\log_{10}$ (*P*-value) of a HWE test on a single SNP. SNPs within sex chromosomes are excluded from HWE tests. Red line indicates the threshold of cut-off value of $-\log_{10}(P)$ of 4, and SNPs above this red line were removed from association tests.

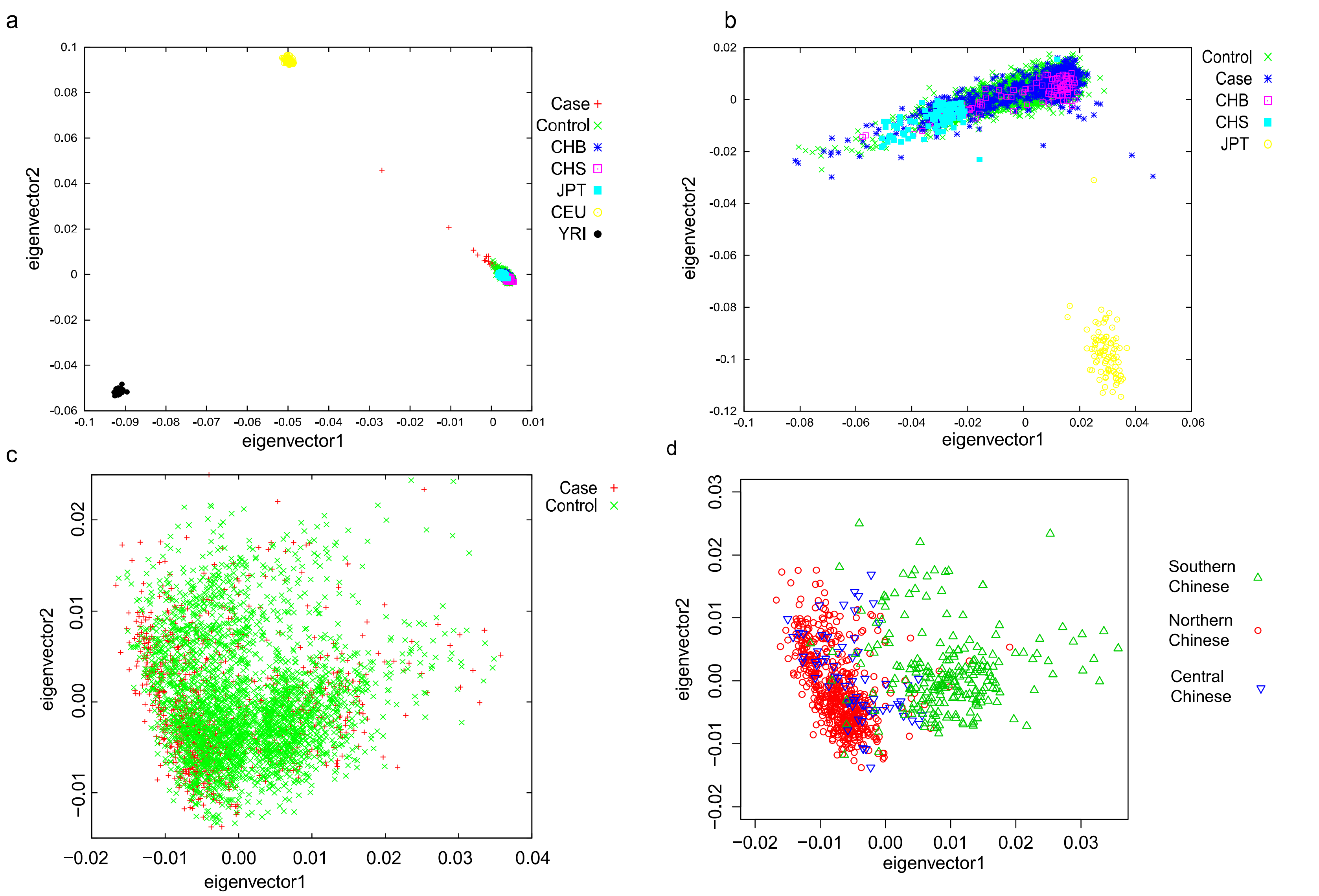

Supplementary Fig.12 Principle component analysis (PCA) used to identify population stratification with data from 939 cases, 2,020 controls and 1KG project. We downloaded the data of YRI (Yoruba in Ibadan, Nigeria), CEU (Utah residents with ancestry from Northern and Western Europe), JPT (Japanese in Tokyo, Japan), CHB (Han Chinese in Beijing, China), and CHS (Han Chinese South, China) from 1KG project website at ftp://ftp.1000genomes.ebi.ac.uk/vol1/ftp/release. We removed variants of INDEL and allele type of AT or CG for strand flip problem before data sets combination. Then we performed PCA on data set of cases, controls, and 1KG project (a), cases, controls, and Asian populations from 1KG project (b), cases and controls (c), and cases only (d). We categorized our samples into northern, central, and southern Chinese according to Xu et al[1].

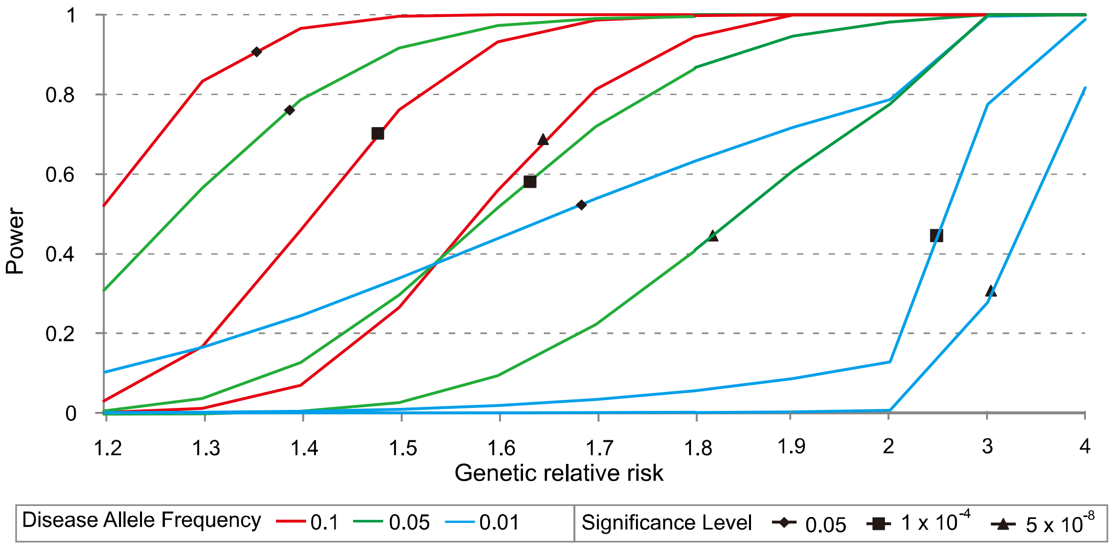

Supplementary Fig.13 Powers of genetic tests for detecting significant associated SNPs with the samples size in this study. X-axis shows the genetic relative risk. Y-axis shows the estimated powers of the sample size with various disease allele frequencies and significance levels.

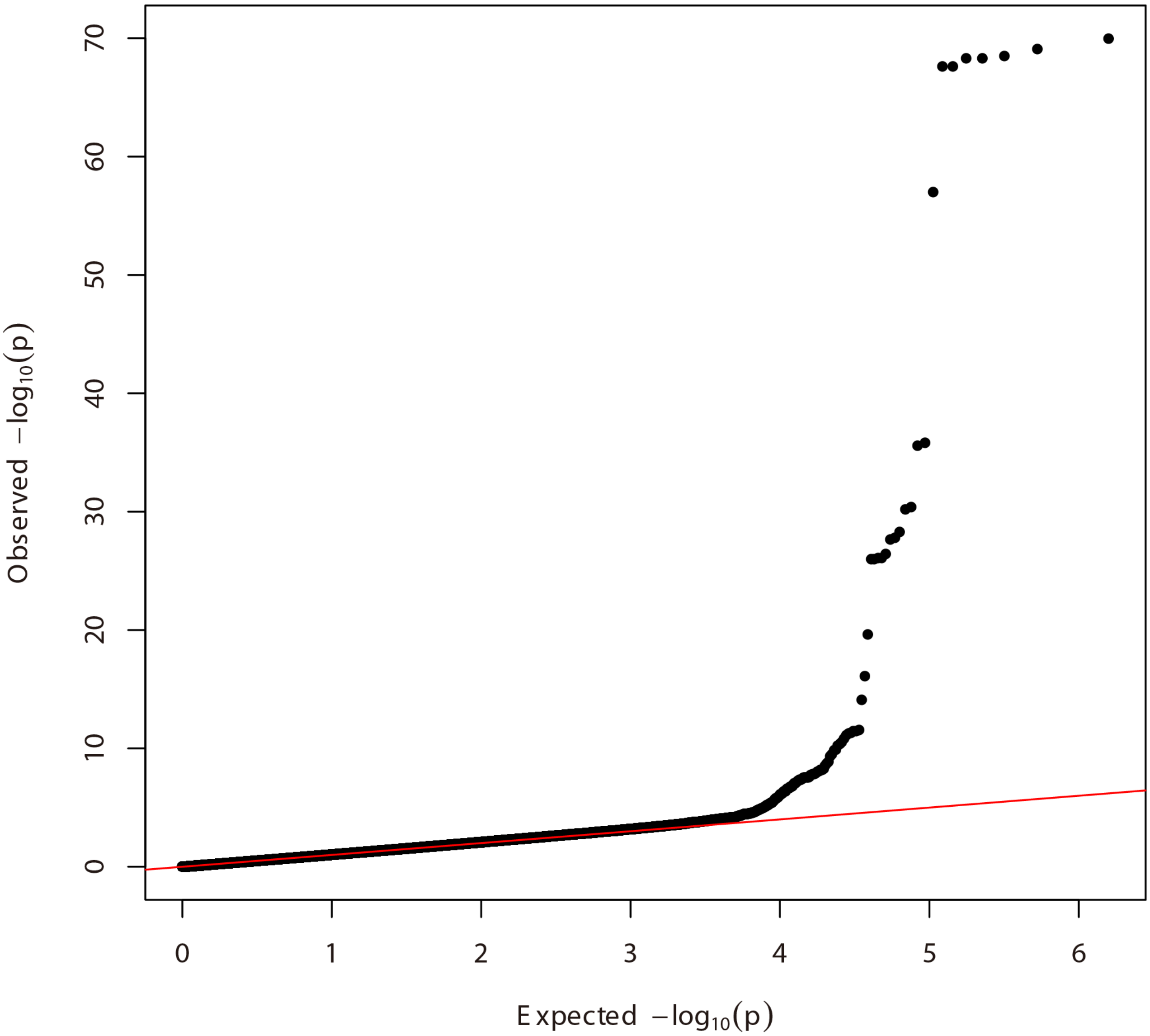

Supplementary Fig.14 Q-Q plot of genome-wide association study on craniofacial microsomia. The quantile-quantile plot of the expected and observed P-values is shown. The black dots represent the observed data, and the red line is the expectation under the null hypothesis of no association. Data include are those passing our final QC.

Supplementary Reference

1. Xu, S. *et al*. Genomic dissection of population substructure of Han Chinese and its implication in association studies. *Am J Hum Genet* **85**, 762-74 (2009).
